# Supplementary material for: Saccharomyces boulardii (CNCM I-745) improves intestinal damage in sepsis by remodeling intestinal flora structure
Source: Front Cell Infect Microbiol. 2025 Jul 31;15:1602792. doi: 10.3389/fcimb.2025.1602792 (PMC12350329; doi:10.3389/fcimb.2025.1602792)
Supplement: Supplementary file 1 [file DataSheet1.zip › summary/summary/7_Differential_analysis/2_LEfSe_result/Group_BW4_vs_AW4_0.05_3.0/LEfSe_Group_BW4_vs_AW4.cladogram.pdf]

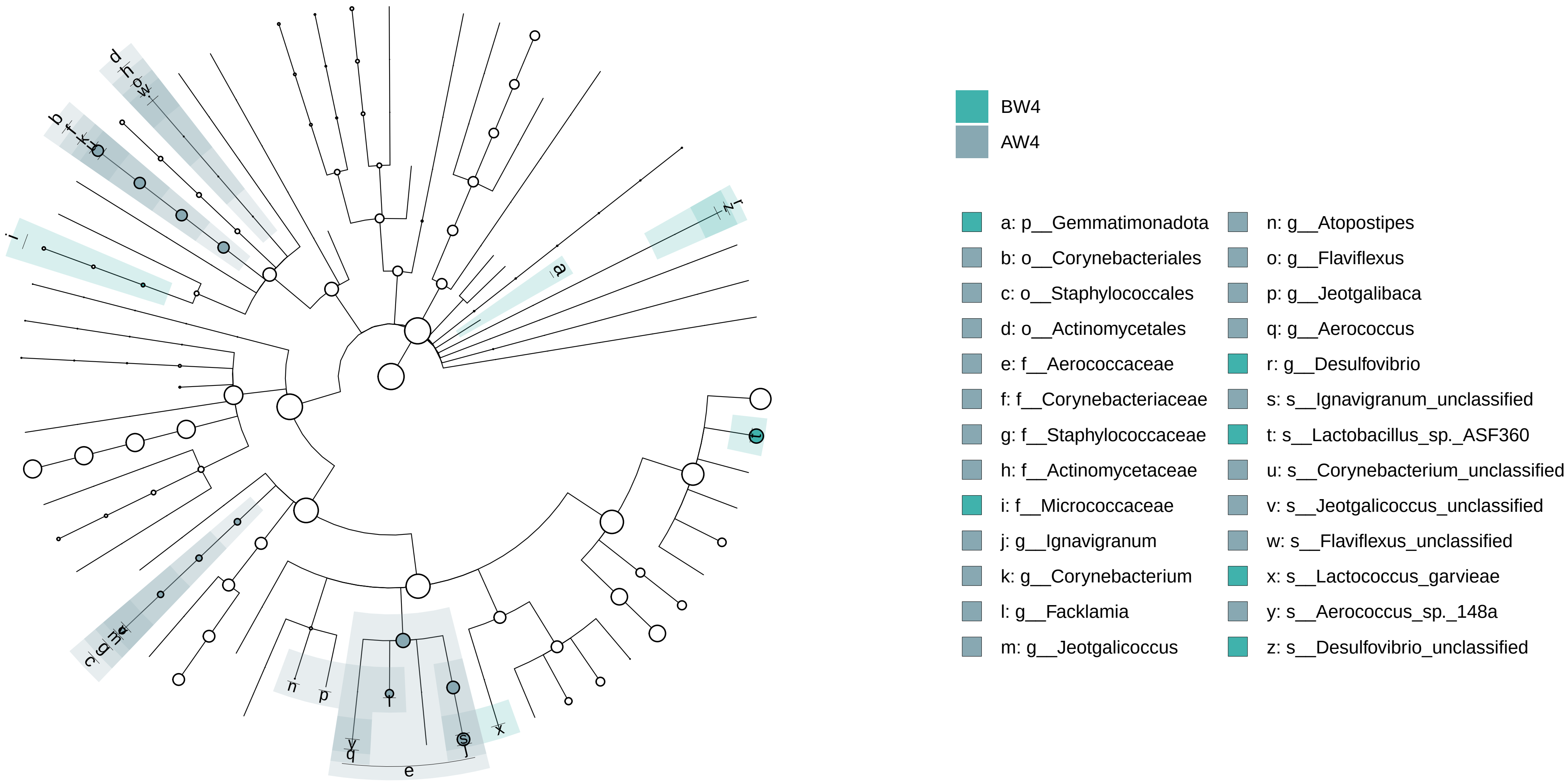

BW4
AW4
a: p__Gemmatimonadota
b: o__Corynebacteriales
c: o__Staphylococcales
d: o__Actinomycetales
e: f__Aerococcaceae
f: f__Corynebacteriaceae
g: f__Staphylococcaceae
h: f__Actinomycetaceae
i: f__Micrococcaceae
j: g__Ignavigranum
k: g__Corynebacterium
l: g__Facklamia
m: g__Jeotgalicoccus
n: g__Atopostipes
o: g__Flaviflexus
p: g__Jeotgalibaca
q: g__Aerococcus
r: g__Desulfovibrio
s: s__Ignavigranum_unclassified
t: s__Lactobacillus_sp._ASF360
u: s__Corynebacterium_unclassified
v: s__Jeotgalicoccus_unclassified
w: s__Flaviflexus_unclassified
x: s__Lactococcus_garvieae
y: s__Aerococcus_sp._148a
z: s__Desulfovibrio_unclassified
